# Latent Fingermark Imaging by Single-Metal Deposition of Gold Nanoparticles and Surface Enhanced Raman Spectroscopy

**DOI:** 10.3389/fchem.2019.00440

**Published:** 2019-06-13

**Authors:** Gitanjali Kolhatkar, Cédric Parisien, Andreas Ruediger, Cyril Muehlethaler

**Affiliations:** ^1^Nanophotonics and Nanoelectronics Group, Institut National de la Recherche Scientifique Énergie-Matériaux-Télécommunication, Varennes, QC, Canada; ^2^Department of Chemistry, Biochemistry, and Physics, Université du Québec à Trois-Rivières, Trois-Rivières, QC, Canada; ^3^Laboratoire de Recherche en Criminalistique, Université du Québec à Trois-Rivières, Trois-Rivières, QC, Canada

**Keywords:** surface enhanced Raman spectroscopy, latent fingermarks, gold nanoparticles, single metal deposition, gold luminescence, chemical composition

## Abstract

In forensic science, there is a high demand for a technique that allows the revelation of fingermarks invisible to the naked eye as well as the chemical information they contain. Here, we present a feasibility study consisting of using both the luminescence enhanced by surface plasmon of gold nanoparticles, and the surface enhanced Raman spectroscopy signal of fingermark chemical components to image latent fingermarks. A latent fingermark deposited on a transparent glass substrate was visually revealed using single-metal deposition employing gold nanoparticles. The resulting enhanced luminescence was monitored over a developed area of the latent fingermark, displaying light regions of 200–400 μm, corresponding to the fingermark ridges. The Raman signal of the fingermark's chemical components was enhanced into a measurable signal. Imaging those Raman peaks revealed the ridges pattern, attesting to the potential of our method. Since SMD is an end-of-sequence revelation technique for which further enhancement techniques do not exist, this work aims at demonstrating the feasibility of the technique in order to apply it on different systems, able to illuminate a complete surface of a few cm, and thus capable of both detecting contaminants in LFM and imaging features of the size of a complete LFM.

## Introduction

Human fingerprints display patterns that are highly selective to each and every individual and remain unchanged throughout their lives, offering an unequaled capacity for identification (Champod et al., 2016). This unique identifier is a powerful tool in forensic science. Fingerprints are composed of friction ridges that are separated into a three level hierarchical order (Jain et al., 2007; Champod et al., 2016). Level 1 refers to the general pattern, Level 2, to minutiae points i.e., endings or bifurcations, and Level 3 includes dimensional attributes like sweat pores and the ridges contours (Hutchins et al., 2013; Champod et al., 2016). Upon contact with an item or a surface with unprotected hands, a person will deposit an image of their fingerprint patterns, referred to as a fingermark (FM), mostly composed of biological secretions and environmental contaminants. Since such FM is mostly not visible, they are then referred to as latent fingermarks (LFM). LFMs are usually not visible to the naked eye and need to be enhanced for forensic applications (Wei et al., 2016).

The available fingermark development techniques target skin secretions from the eccrine, apocrine and sebaceous glands (Wei et al., 2016). Eccrine secretions are provided by glands located all over the body that can be found in higher density on the palms of the hands and the sole of the feet. They are water-based solutions containing traces of organic compounds and inorganic salts. The apocrine glands can be found in areas such as the armpits and the hair and excrete a viscous milky fluid composed of proteins, carbohydrates, cholesterol, iron, and steroid sulfates. Finally, the sebaceous glands, found in the dermis layer, are associated with body hair and secrete sebum fluids consisting of waxes, squalene and saturated fats (Holder et al., 2011). Those secretions are known as endogenous factors and provide the biological characteristics of an individual, while external contaminations (i.e., cosmetics, food residues) are referred to as exogenous composition factors (Cai et al., 2017). Therefore, in addition to providing the patterns and enabling the identification of an individual, the development of fingermarks can offer information regarding the environment and lifestyle of a person. Indeed, the presence of gunpowder or drugs have been extracted from LFMs (Day et al., 2004; Charlton et al., 2013; Groeneveld et al., 2015; Figueroa et al., 2017).

Several revelation techniques have been explored that exploit the specific binding to skin secretions to provide the contrast. Black powder, cyanoacrylate fuming, and amino acid reagents (Ninhydrin, Indanedione Zinc), are the most favored revelation methods providing the necessary versatility to detect traces on non-porous and porous surfaces. The development techniques are based on a chemical enhancement to increase contrast, but do not allow for the identification of specific endogenous or exogenous components (Lennard, 2014). Specific LFM-related components were monitored using label-free mass spectrometry (MS) detection methods (Musah et al., 2012), such as desorption electrospray ionization (DESI) (Ifa et al., 2008), matrix-assisted laser desorption ionization (MALDI) (Wolstenholme et al., 2009; Bradshaw et al., 2011; Groeneveld et al., 2015), and time-of-flight secondary ion mass spectrometry (TOF-SIMS) (Bailey et al., 2010; Hinder and Watts, 2010; Cai et al., 2017). The photoluminescence of nanoparticles (NPs) such as ZGO:Ga, Mn-ConA (Wang et al., 2017), or that of upconversion NPs with a lysozyme-bonding aptamer (Wang et al., 2014) was exploited to image fingermarks. Successful revelation was also achieved through FTIR (Tahtouh et al., 2005; Ricci et al., 2007), and Raman spectroscopy (Day et al., 2004; Widjaja, 2009; Deng et al., 2012). However, MS techniques are destructive while Raman spectroscopy and other vibrational techniques require long measurement times due to the weak signal of the chemical compounds. This weak signal can also be overshadowed by a substrate background (Figueroa et al., 2017).

To overcome this detection limit and reduce the measurement time, surface enhanced Raman spectroscopy (SERS) has been proposed (Connatser et al., 2010; Cialla et al., 2012; Song et al., 2012; Muehlethaler et al., 2016). This powerful surface-sensitive technique exploits the plasmonic properties of metallic nanostructures, most commonly silver (Ag) or gold (Au), to amplify a Raman signal. This enhancement is obtained by exciting the localized surface plasmon (LSP) of the metallic nanostructures through the collective coherent oscillations of free electrons in a continuous band structure. This LSP has a resonance, known as localized surface plasmon resonance (LSPR), which will enhance a near-field signal that was initially too weak to be detected and make it measurable. Additionally, metallic NPs display a strong luminescence originating from interband transitions caused by the direct radiative recombination of Fermi level electrons with holes from the sp or d bands (Mooradian, 1969). This luminescence will be enhanced by the LSPR, resulting in a measurable emission signal (Mohamed et al., 2000). This technique was employed to image fingermarks by imaging the signal of proteins, enhanced using an antibody Raman probe (Song et al., 2012). Yet, proteins are only a minor component of fingerprint secretions compared to free amino acids or lipids, making it challenging to detect on conventional traces. Besides, revelation techniques exploiting gold and silver NPs, known as multi-metal deposition (MMD) and single-metal deposition (SMD) approaches were introduced in LFM sequences of revelation (Becue et al., 2007; Stauffer et al., 2007; Fairley et al., 2012; Newland et al., 2016). These two-steps techniques consist in using metal NP, either gold (Au) or silver (Ag) to reveal a fingermark independently from its chemical composition and from the substrate porosity. They combine an important sensitivity to a high selectivity, as the NPs will preferentially attach on the fingermark compounds (Mohamed, 2011). The most important advantage of these methods is the universality with which they allow detection on non-porous, semi-porous and porous surfaces. Yet, while MMD and SMD enhance the visual contrast, it is still not sufficient for these techniques to become standard procedures. While the few limitations are continuously being improved (Becue et al., 2012), the nanoparticle layer generally precludes any additional technique to be used, and SMD/MMD are generally seen as end-of-sequence techniques. However, the NPs size, in the 1–100 nm range, is well-adapted for SERS application and likely to provide strong plasmonic resonance and luminescence (Muehlethaler et al., 2016). The possibility to visualize a chemical map of the fingermark might be valuable on difficult substrates (colored or dark), or for poorly revealed traces.

This work demonstrates the potential of combining SMD to SERS imaging to reveal the fingermark patterns. Gold NPs are deposited on a glass substrate presenting a LFM and their luminescence is monitored to reproduce the ridges patterns. In addition, the signal of chemicals composing the LFM, rendered measurable through a SERS effect, is imaged to reconstruct the fingermark. While most studies so far exploited immunoassays with antigen-antibody functionalized probes to produce a SERS signal, our method exploits the nanoparticles-enhancing electromagnetic field to create a chemical contrast with the substrate. By doing so, the substrate contribution is removed, allowing visualization of precise fingermark details with high resolution, irrespective of the substrate color. This simple, non-destructive approach does not require any reagent, and offers an added visualization possibility without any modification of the original SMD revelation. In addition, this approach provides a way to perform fast non-destructive measurements that reveal the LFM patterns as well as endogenous and exogenous chemical information.

## Materials and Methods

### Sample Preparation

The sample consists of standard microscopy glass slides bearing a latent fingermark revealed through SMD (Becue et al., 2007; Stauffer et al., 2007). Natural fingermarks were deposited by the donors, which were asked not to wash their hands for 30 min prior to deposition, and to slightly rub their hands together to ensure distribution of both eccrine and sebaceous secretions. A single depletion was made directly on the glass slide. The revelation was performed by immersing the sample in a succession of baths. First, it was dipped for 2–3 min in H_2_O for rinsing to remove undesired contaminations. Second, in a gold nanoparticles (AuNPs) solution. The AuNPs (15–20 nm in diameter) were formed from a prior HAuCl_4_ and trisodium citrate dihydrate synthesis. The colloids preferentially attach on the fingermark ridges under precise pH conditions (2.5–2.8), which need be controlled. Third, it was rinsed in H_2_O to remove the NP that may have attached on the glass substrate itself. It was then plunged in a second solution of HAuCl_4_ and hydroxylamine hydrochloride (HONH_2_-HCl) which contributes to reinforcement over the gold already present on the ridges before being rinsed in H_2_O (Stauffer et al., 2007). Lastly, the sample was dried in ambient conditions.

### SERS Measurements

The luminescence and SERS measurements were performed using linearly polarized TEM_00_ He-Ne laser operating at 632.8 nm and an AIST-NT OmegaScope 1000 equipped with a thermoelectrically cooled CCD detector and Nanofinder 30 Raman spectrometer. The laser beam was focused on the sample through a 0.28 NA Mitutoyo MPlan Apo 10 × objective. A laser power of 0.31 mW was used throughout the measurements. A typical spectrum at one pixel point consists of one accumulation of 0.5–1 s integration time. No further post-treatments were realized on the spectra.

## Results and Discussion

**Figure 1:**
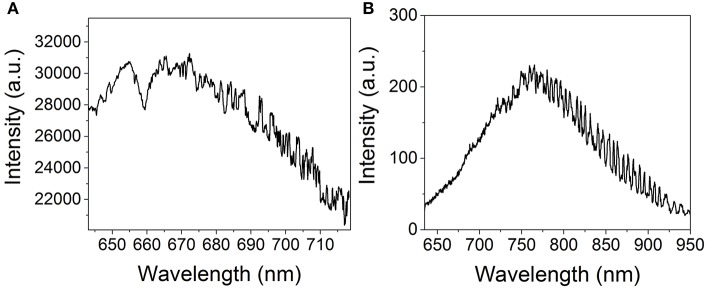
Typical luminescence spectra of **(A)** 15 nm gold nanoparticles and **(B)** the glass substrate on which the fingerprint was revealed.

**Figure 2:**
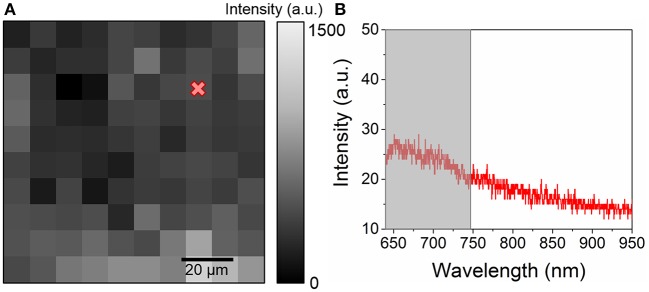
**(A)** 100 ×100 μm^2^ map of the integrated intensity between 640 and 750 nm acquired with a 5 μm spatial resolution on a reference sample consisting of a fingermark on a glass cover slide with no gold NP and **(B)** example of a spectrum obtained from the map. The gray region indicates the integrated intensity range.

The typical luminescence spectra of gold NPs and the glass substrate are presented in Figure 1. The glass substrate depicts a wide Gaussian-shaped luminescence centered at ~800 nm. The gold NPs display a relatively narrow Lorentzian-shaped luminescence centered at ~670 nm. This signal corresponds to the luminescence enhanced by the surface plasmon (Zheng et al., 2012). While this luminescence shifts with the radius of the NP, both the glass substrate and the NP luminescence remain in different spectral ranges and can therefore be distinguished. In both cases, the noise observed at higher wavelengths is due to optical etaloning in the CCD detector. Neither of those spectra display any Raman peaks. The luminescence signal was first mapped over a reference sample consisting of a glass cover slide bearing a fingermark and no gold NPs. The map of the integrated intensity (Figure 2A) appears homogeneous, with no regions displaying significantly higher intensities. The spectra (Figure 2B) exhibit a weak luminescence all over the mapped region. This signal can be attributed to the cover slide.

**Figure 3:**
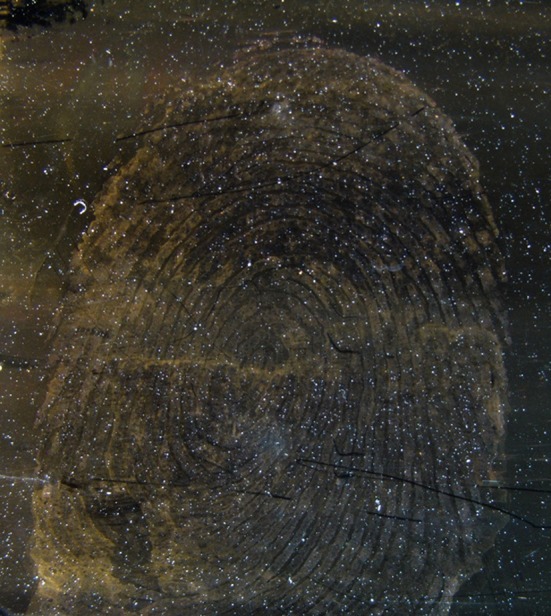
Stereomicroscopy image (5 × objective) of a complete SMD revealed fingermark on a glass substrate.

**Figure 4:**
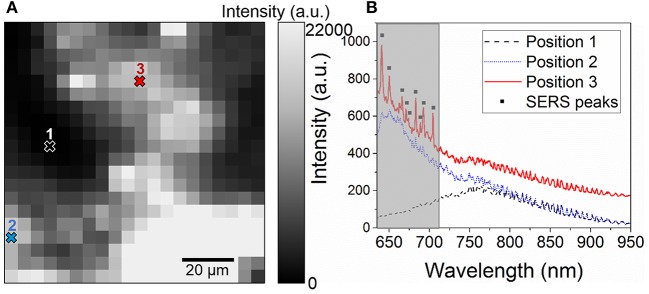
**(A)** 100 × 100 μm^2^ map of the integrated intensity in the 640–710 nm performed on a LFM revealed with gold NPs with a 5 μm spatial resolution and **(B)** Raman spectra acquired at three different positions of the map showing the spectrum of glass (Position 1), a region of the fingerprint with gold NPs (Position 2), and a region with SERS enhancement (Position 3, black squares). The gray region indicates the integrated intensity range.

Fingermarks revealed by SMD on glass substrates in illustrated in Figure 3. After the revelation, the ridge are well-defined as indicated by the golden-brown regions with limited background contamination. 100 ×100 μm map of the intensity of the luminescence signal was acquired on a revealed area of the fingermark with a 5 μm spatial resolution, as presented in Figure 4A. The light region corresponds to areas covered with gold NPs while the dark regions are attributed to the glass only, as confirmed by the spectra illustrated in Figure 4B. The intensities recorded on the map presents a strong gold luminescence, much higher than that of the glass substrate. A SERS effect is also indicated by the presence of Raman peaks on the gold luminescence spectrum at Position 3 (red curve). The peak positions are provided in Table 1. They can be linked to functional groups of organic molecules, mainly proteins, lipoproteins, DNA, and aminoacids, as listed in Table 1. This is coherent with the chemicals produced by the eccrine, apocrine and sebaceous glands, that are expected to remain after the SMD treatment. Such peaks were not observed on the reference sample presented in Figure 2, confirming that the signal is due to an enhancement effect originating from the presence of gold NPs on the fingermark ridges and not to the fingermark alone.

**Table 1:** Raman peaks recorded on the revealed fingermark and their tentative assignment.

**Peak position (cm^−1^)**	**Tentative assignment (Movasaghi et al., 2007)**
197	N/A
422	Symmetric stretching ${\text{Po}}_{4}^{3-}$
788	Nucleic acid measure
900	C-O-C sugar skeletal
952	CH_3_ stretching (proteins α-helix)
1,162	Quinoid ring
1,284	Amide III and CH_2_ wagging (glycine + proline)
1,361	Guanine/tryptophan
1,606	Cytosine (NH2), Ring C-C stretch of phenyl, Phenylalanine, tyrosine, C55C (protein)

Furthermore, the trace revealed in Figure 4A is not uniform, and is much narrower than a typical fingermark ridge (200–400 μm), revealing that the gold NPs tend to form clusters instead of attaching homogeneously on the fingermark ridges. This also suggests that the fingermark chemical constituents were not deposited uniformly on the glass substrate, adding an uncertainty on the revelation. Nevertheless, this indicates that by monitoring the gold luminescence as well as the SERS peaks, the fingermark can be revealed. Furthermore, even in the regions with a strong gold luminescence and a SERS effect, the signal of the glass substrate remains visible. Therefore, the glass spectrum can be used to normalize the spectra for the LFM revelation.

**Figure 5:**
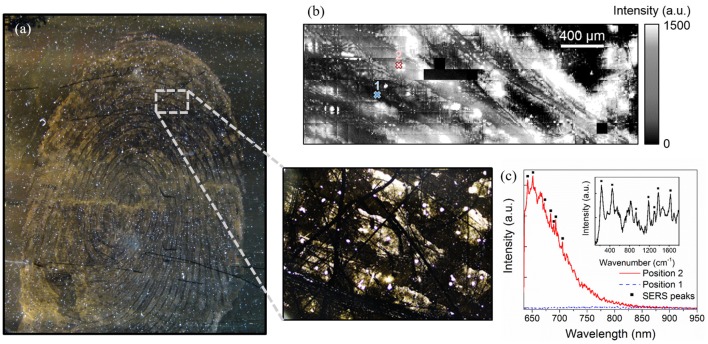
**(a)** optical dark-field microscopy image (4 × objective) of the sample showing three fingermark ridges (golden-brown regions) deposited on a glass substrate (black region), **(b)** 1,100 × 3,100 μm^2^ integrated intensity map of the gold luminescence acquired on a region of the fingerprint revealed with gold NP with a spatial resolution of 10 μm, **(c)** Raman spectra taken in a dark region (Position 1) and on a high intensity region (Position 2), revealing SERS peaks (black squares). The inset presents the Raman spectrum obtained at Position 2 after subtracting the gold contribution. All spectra were normalized using the spectrum of the glass substrate.

To reconstruct the fingermark pattern (Figure 5a), the gold luminescence was mapped over a large region (1,100 × 3,100 μm^2^) of the LFM revealed with gold NPs, as illustrated in Figure 5b. These dimensions were chosen to display 2–3 ridges. To eliminate intensity variations due to the substrate flatness and changes in the focus, and assuming that the glass signal should remain constant all over the sample surface, the spectra were normalized with respect to the glass luminescence.

The image obtained by this process presents light and dark regions. The dark regions (position 1, Figure 5b) display a very low intensity while the light regions (position 2, Figure 5b) present a strong gold luminescence signal. The light regions have a width of 200–400 μm, which corresponds to the typical width of ridges. The pattern observed in Figure 5b can be correlated with the optical microscopy image acquired in the same region (Figure 5a, zoomed-in), where the fingermark revealed by SMD on the glass substrate shows a typical reddish-brown coloration due to the presence of gold NPs on the ridges. We can see that these patterns are not always well-defined, and gold aggregates can be observed between the ridges. This could be due to poor transfer conditions such as smearing, over pressure, under-pressure or double tapping. This could also be caused by the revelation process. Indeed, as the revelation is realized through a series of baths, an overexposure of the fingermark to gold NPs can result in a coloring of the substrate. Brown stains are common as a result of the revelation. In addition, the revelation technique can produce dashed or dot-like ridges instead of continuous patterns, such as those observed in Figure 5b (Becue et al., 2012).

Due to the very high spatial resolution used here as compared to the features dimensions, the image presented is very rich in information, which results in the blurring-like effect obtained here. Each spectrum provides very sensitive results of the pixel it represents due to the SERS enhancement. However, black lines appearing in the ridges run over several pixels, confirming that they are caused by the sample and not due to an artifact. Possible causes are fingermark features, scratches in the glass substrate or sample degradation. Therefore, those marks indicate that we are able to reveal substructures of the LFM. Finally, the black pixels are due to a non-optimized system. Our resolution remains limited by the quality of the fingermark and the revelation technique. Nevertheless, this study attests to the potential of this technique for the visualization of LFMs.

**Figure 6:**
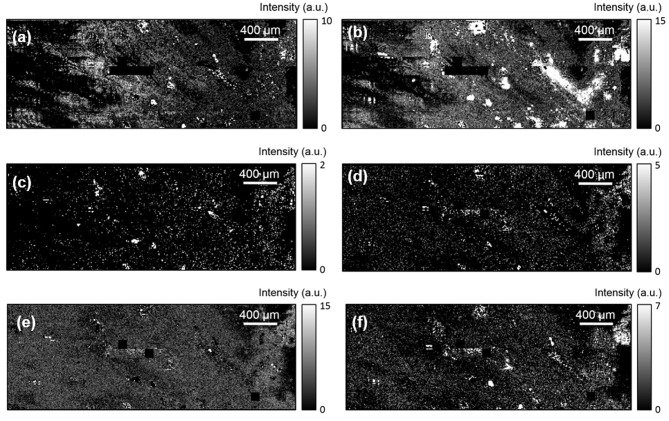
1,100 × 3,100 μm^2^ maps of the SERS peaks located at **(a)** 200 cm^−1^, **(b)** 420 cm^−1^, **(c)** 900 cm^−1^, **(d)** 1,160 cm^−1^, **(e)** 1,360 cm^−1^, and **(f)** 1,600 cm^−1^.

Furthermore, a SERS effect was recorded in the light regions, as shown by the Raman peaks presented in Figure 5c, consistently with that of the high resolution map (Figure 4). The Raman peaks measured here (black squares), located at 200, 420, 900, 1,160, 1,360, and 1,600 cm^−1^ were also observed in Figure 4B. These peaks are not due to etaloning in the CCD. Two peaks measured in Figure 4B are not visible here, most likely due to the lower integration time (0.5 s instead of 1 s). The intensity of the peaks were mapped over the same area as that of Figure 5a in order to image the hot spots. To do so, the gold luminescence was fitted and subtracted from the spectra in order to obtain the Raman peaks only, as shown in Figure 5c, attesting to the accuracy of our fit. The maps of the different Raman peaks are presented in Figure 6.

The first two peaks (Figures 6a,b), located at ~200 and 420 cm^−1^, reveal the most hot spots. Indeed, these two peaks are located close to the maximum of the gold signal, which corresponds to the spectral range enhanced by the LSPR. The maps of these two peaks results in images very similar to that of the gold luminescence. This reveals that the gold luminescence is stronger at the SERS hot spots, and confirms that the gold NPs clusters preferentially form on the secretions.

Regarding the other four Raman peaks' maps (Figures 6c–f), the bright spots are very few, and no evident correlation with the gold luminescence can be seen. Yet, all four maps display similar features, indicating that these four chemical components all attached at the same positions. This further reveals that these components are most likely due to fingerprint secretions and not to contaminations. Therefore, this technique allows us to recover not only the general LFM patterns using the gold luminescence but also by imaging the chemical components via SERS measurements. In addition, correlating the positions of the different chemical components provides us with a way to discriminate contaminants from human secretions.

While it would be interesting to conduct this study on a large region and reveal the complete fingermark, we were not able to perform those measurements due to the limitations of our setup. The map dimension is limited by the stage. In addition, the laser spot employed here has a diameter of ~3 μm. Therefore, the minimum spatial resolution we can use to accurately map the LFMs is ~20 μm, which results in longer acquisition times and heavier data files. By using of a lower spatial resolution we would risk missing the LFM features and the resulting map will not be representative of the mark.

The purpose of this study is to understand the phenomenon and verify that SERS and luminescence effects could be observed at the microscopic scale. It also aims at demonstrating the feasibility of the technique in order to apply it on different systems, able to illuminate a complete surface of a few cm and thus better adapted for the imaging of features of the size of a complete LFM.

Nevertheless, this study paves the way for LFM studies in forensic science. By combining SMD revelation to SERS measurements, we can visualize the fingermark through gold luminescence and chemically through the secretions' composition and/or contaminants. Also drugs metabolites might be reflected in the SERS signal. Therefore, the SERS effect could potentially be used to identify contaminants such as gun powder residues or drugs, which will be explored in future studies.

## Conclusion

This paper demonstrates the potential of combining SMD treatments with SERS imaging to reconstruct LFMs. Using gold NPs and the SMD method, the contrast of a LFM deposited on a glass substrate is enhanced to reveal it visually. By monitoring the gold luminescence enhanced by LSPR, we are able to reproduce ridges patterns. In addition, Raman peaks measured on these ridges suggest a SERS enhancement due to the presence of gold NPs. These Raman peaks can be attributed to chemicals produced by the apocrine, eccrine, and sebaceous glands that are not removed by the SMD treatment. Comparison with a reference sample consisting in an unrevealed LFM deposited on a glass cover slide confirms the SERS nature of the signal. By imaging the SERS peaks over the surface of the revealed fingermark, we are able to reconstruct the LFM. Also, correlating different chemical components provides us with a way to discriminate contaminants. The method proposed here will be applied to other systems, better adapted for larger scale imaging, to reveal a complete LFM. This methodology will prove useful for contrast enhancement of LFM on difficult substrates and a combined identification of fingermarks' secretions and/or contaminants. SERS imaging through the gold nanoparticles provides an additional visualization technique for SMD, that is usually an end-of-sequence revelation technique for which further enhancement techniques do not exist.

## Author Contributions

GK, CP, AR, and CM designed the experiments and were responsible for the results analysis and their interpretation. GK and CP performed the measurements and the data treatment and co-wrote the paper. The project was supervised by CM.

### Conflict of Interest Statement

The authors declare that the research was conducted in the absence of any commercial or financial relationships that could be construed as a potential conflict of interest. The handling editor declared a past co-authorship with one of the authors CM.

## References

[B1] Bailey M. J. Jones B. N. Hinder S. Watts J. Bleay S. Webb R. P. (2010). Depth profiling of fingerprint and ink signals by SIMS and MeV SIMS. Nucl. Instrum. Methods Phys. Res. Sect. B 268, 1929–1932. 10.1016/j.nimb.2010.02.104

[B2] Becue A. Champod C. Margot P. (2007). Use of gold nanoparticles as molecular intermediates for the detection of fingermarks. Forensic Sci. Int. 168, 169–176. 10.1016/j.forsciint.2006.07.014 16920302

[B3] Becue A. Scoundrianos A. Moret S. (2012). Detection of fingermarks by colloidal gold (MMD / SMD)—beyond the pH 3 limit. Forensic Sci. Int. 219, 39–49. 10.1016/j.forsciint.2011.11.024 22230765

[B4] Bradshaw R. Wolstenholme R. Blackledge R. D. Clench M. R. Ferguson L. S. Francese S. (2011). A novel matrix-assisted laser desorption/ionisation mass spectrometry imaging based methodology for the identification of sexual assault suspects. Rapid Commun. Mass Spectrom. 25, 415–422. 10.1002/rcm.4858 21213360

[B5] Cai L. Xia M. C. Wang Z. Zhao Y. B. Li Z. Zhang S.. (2017). Chemical visualization of sweat pores in fingerprints using GO- enhanced TOF-SIMS. Anal. Chem. 89, 8372–8376. 10.1021/acs.analchem.7b01629 28700825

[B6] Champod C. J. Lennard C. Margot P. Stoilovic M. (2016). Fingerprints and Other Ridge Skin Impressions. Boca Raton, FL: CRC press 10.1201/b20423

[B7] Charlton D. T. Bleay M. Sears V. G. (2013). Evaluation of the multimetal deposition process for fingermark enhancement in simulated operational environments. Anal. Methods 5, 5411–5417. 10.1039/c3ay40533h

[B8] Cialla D. März A. Böhme R. Theil F. Weber K. Schmitt M.. (2012). Surface-enhanced Raman spectroscopy (SERS): progress and trends. Anal. Bioanal. Chem. 403, 27–54. 10.1007/s00216-011-5631-x 22205182

[B9] Connatser R. M. Prokes S. M. Glembocki O. J. Schuler R. L. Gardner C. W. Lewis S. A.. (2010). Toward surface-enhanced raman imaging of latent fingerprints. J. Forensic Sci. 55, 1462–1470. 10.1111/j.1556-4029.2010.01484.x 20629909

[B10] Day J. S. Edwards H. G. Dobrowski S. A. Voice A. M. (2004). The detection of drugs of abuse in fingerprints using Raman spectroscopy I: latent fingerprints. Spectrochim. Acta Part A Mol. Biomol. Spectrosc. 60, 563–568. 10.1016/S1386-1425(03)00263-4 14747080

[B11] Deng S. Liu L. Liu Z. Shen Z. Li G. He Y. (2012). Line-scanning Raman imaging spectroscopy for detection of fingerprints. Appl. Opt. 51, 3701–3706. 10.1364/AO.51.003701 22695646

[B12] Fairley C. Bleay S. M. Sears V. G. Nicdaeid N. (2012). A comparison of multi-metal deposition processes utilising gold nanoparticles and an evaluation of their application to ‘low yield’ surfaces for finger mark development. Forensic Sci. Int. 217, 5–18. 10.1016/j.forsciint.2011.09.018 22030482

[B13] Figueroa B. Chen Y. Berry K. Francis A. Fu D. (2017). Label-free chemical imaging of latent fingerprints with stimulated raman scattering microscopy. Anal. Chem. 89, 4468–4473. 10.1021/acs.analchem.6b04213 28322553

[B14] Groeneveld G. de Puit M. Bleay S. Bradshaw R. Francese S. (2015). Detection and mapping of illicit drugs and their metabolites in fingermarks by MALDI MS and compatibility with forensic techniques. Sci. Rep. 5, 11716. 10.1038/srep11716 26118853 PMC4484357

[B15] Hinder S. J. Watts J. F. (2010). SIMS fingerprint analysis on organic substrates. Surf. Interface Anal. 42, 826–829. 10.1002/sia.3497

[B16] Holder E. H. Robinson L. O. Laub J. H. (2011). The Fingerprint Sourcebook. Washington, DC: U.S. Dept. of Justice, Office of Justice Programs, National Institute of Justice.

[B17] Hutchins L. A. States U. Service S. (2013). Identification and Classification, Encyclopedia of Forensic Sciences 2nd Edn, eds Siegel J. Saukko P. (Waltham: Elsevier Inc.; Academic Press).

[B18] Ifa D. R. Manicke N. E. Dill A. L. Cooks R. G. (2008). Latent fingerprint chemical imaging by mass spectrometry. Science. 321, 805. 10.1126/science.1157199 18687956

[B19] Jain A. K. Chen Y. Demirkus M. (2007). Pores and ridges : high-resolution fingerprint matching using level 3 features. IEEE Trans. Pattern Anal. Mach. Intell. 29, 15–27. 10.1109/TPAMI.2007.250596 17108380

[B20] Lennard C. (2014). Fingermark detection and identification : current research efforts. Aust. J. Forensic Sci. 46, 293–303. 10.1080/00450618.2013.839743

[B21] Mohamed A. A. (2011). Gold is going forensic. Gold Bull. 44, 71–77. 10.1007/s13404-011-0013-x

[B22] Mohamed M. B. Volkov V. Link S. El-Sayed M. A. (2000). The ‘lightning’ gold nanorods: fluorescence enhancement of over a million compared to the gold metal. Chem. Phys. Lett. 317, 517–523. 10.1016/S0009-2614(99)01414-1

[B23] Mooradian A. (1969). Photoluminescence of metals. Phys. Rev. Lett. 22, 185–187. 10.1103/PhysRevLett.22.185

[B24] Movasaghi Z. Rehman S. Rehman I. (2007). Raman Spectroscopy of biological tissues. Appl. Spectrosc. Rev. 42, 493–541. 10.1080/05704920701551530

[B25] Muehlethaler C. Leona M. Lombardi J. R. (2016). Review of surface enhanced raman scattering applications in forensic science. Anal. Chem. 88, 152–169. 10.1021/acs.analchem.5b04131 26638887

[B26] Musah R. A. Cody R. B. Dane A. J. Vuong A. L. Shepard J. R. (2012). Direct analysis in real time mass spectrometry for analysis of sexual assault evidence. Rapid Commun. Mass Spectrom. 26, 1039–1046. 10.1002/rcm.6198 22467453

[B27] Newland T. G. Moret S. Bécue A. Lewis S. W. (2016). Further investigations into the single metal deposition (SMD II) technique for the detection of latent fingermarks. Forensic Sci. Int. 268, 62–72. 10.1016/j.forsciint.2016.09.004 27693827

[B28] Ricci C. Bleay S. Kazarian S. G. (2007). Spectroscopic imaging of latent fingermarks collected with the aid of a gelatin tape. Anal. Chem. 79, 5771–5776. 10.1021/ac070580j 17602672

[B29] Song W. Mao Z. Liu X. Lu Y. Li Z. Zhao B.. (2012). Detection of protein deposition within latent fingerprints by surface-enhanced Raman spectroscopy imaging. Nanoscale 4, 2333–2338. 10.1039/c2nr12030e 22371039

[B30] Stauffer E. Becue A. Singh K. V. Thampi K. R. Champod C. Margot P. (2007). Single-metal deposition (SMD) as a latent fingermark enhancement technique : an alternative to multimetal deposition (MMD). Forensic Sci. Int. 168, e5–e9. 10.1016/j.forsciint.2006.12.009 17275233

[B31] Tahtouh M. Kalman J. R. Roux C. Lennard C. Reedy B. J. (2005). The detection and enhancement of latent fingermarks using infrared chemical imaging. J. Forensic Sci. 50, 64–72. 10.1520/JFS2004213 15830998

[B32] Wang J. Ma Q. Liu H. Wang Y. Shen H. Hu X.. (2017). Time-gated imaging of latent fingerprints and specific visualization of protein secretions via molecular recognition. Anal. Chem. 89, 12764–12770. 10.1021/acs.analchem.7b03003 29111687

[B33] Wang J. Wei T. Li X. Zhang B. Wang J. Huang C.. (2014). Near-infrared-light-mediated imaging of latent fingerprints based on molecular recognition. Angew. Chem. Int. Ed. 53, 1616–1620. 10.1002/anie.201308843 24452926

[B34] Wei Q. Zhang M. Ogorevc B. Zhang X. (2016). Recent advances in the chemical imaging of human fingermarks (a review). Analyst 141, 6172–6189. 10.1039/C6AN01121G 27704072

[B35] Widjaja E. (2009). Latent fingerprints analysis using tape-lift, Raman microscopy, and multivariate data analysis methods. Analyst 134, 769–775. 10.1039/B808259F 19305929

[B36] Wolstenholme R. Bradshaw R. Clench M. R. Francese S. (2009). Study of latent fingermarks by matrix-assisted laser desorption/ionisation mass spectrometry imaging of endogenous lipids. Rapid Commun. Mass Spectrom. 23, 3031–3039. 10.1002/rcm.4218 19711300

[B37] Zheng J. Zhou C. Yu M. Liu J. (2012). Different sized luminescent gold nanoparticles. Nanoscale 4, 4073–4083. 10.1039/c2nr31192e 22706895 PMC3404491

